# Supplementary material for: An integrated multimodal model of alcohol use disorder generated by data-driven causal discovery analysis
Source: Commun Biol. 2021 Mar 31;4:435. doi: 10.1038/s42003-021-01955-z (PMC8012376; doi:10.1038/s42003-021-01955-z)
Supplement: Supplementary file 2 — Supplementary Information [file 42003_2021_1955_MOESM2_ESM.pdf]

Supplementary Material for:
An Integrated Multimodal Model of Alcohol Use Disorder Generated by Data-Driven Causal Discovery Analysis

Authors: Eric Rawls, Ph.D.[1], Erich Kummerfeld, Ph.D.[2], and Anna Zilverstand, Ph.D.[1]
Affiliations: [1]Department of Psychiatry and Behavioral Sciences, University of Minnesota, [2]Institute for Health Informatics, University of Minnesota

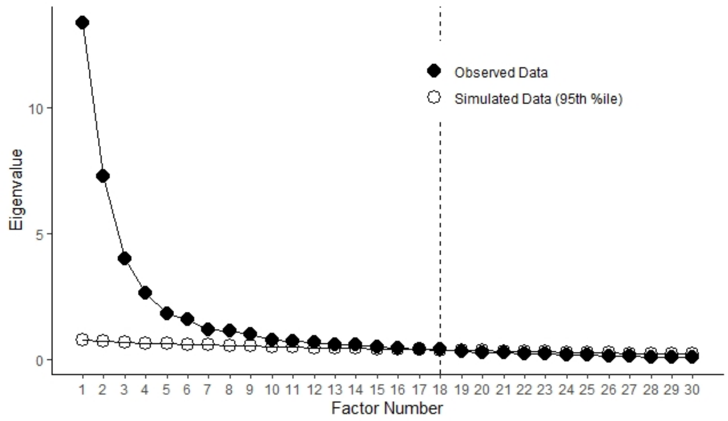

Supplementary Figure S1. Permutation test for eigenvalue significance (parallel analysis).

Supplementary Table S1. Percent variance explained per factor, eigenvalues, and cumulative variance.

Factor	Proportion Variance	Cumulative Variance	Eigenvalue
Somaticism	0.03	0.03	13.36
Fluid Cognition	0.03	0.06	7.28
Internalizing	0.03	0.09	4.02
Gamble RT	0.03	0.12	2.62
Inattention	0.03	0.16	1.81
Visuospatial	0.02	0.19	1.61
Social Support	0.03	0.22	1.2
Processing Speed	0.02	0.24	1.12
Externalizing	0.03	0.27	1.02
Avoidance	0.02	0.29	0.8
Language Task	0.02	0.32	0.74
Relational Task	0.02	0.35	0.69
Delay Discounting	0.02	0.37	0.59
N-Back Task	0.03	0.39	0.58
Negative Affect	0.03	0.41	0.49
CrystalizedIQ	0.03	0.43	0.47
Positive Affect	0.02	0.44	0.41
Agreeableness	0.01	0.46	0.4

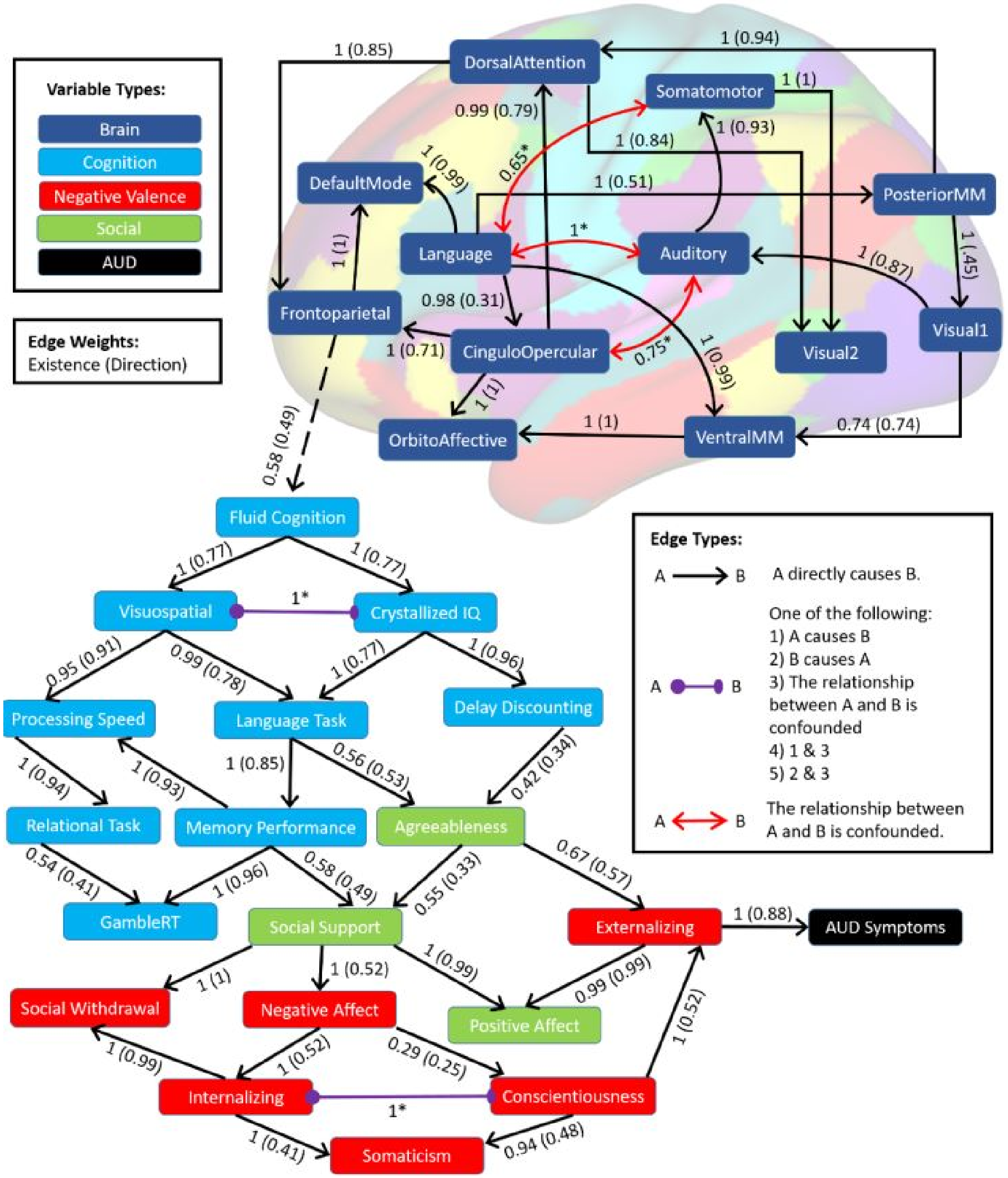

Supplementary Figure S2. Graphical results of jackknife stability analysis. Each directed edge receives two indices of stability: the first is an indication of how often the edge is present at all in jackknifed repetitions (regardless of orientation; 1 being 100% of repetitions), and the second number (in parentheses) is an indication of how often the edge is present and correctly oriented in jackknife repetitions (unoriented edges receive only one index of stability). Note that all edges were significant ($p < .001$) in the SEM; therefore, this analysis does not characterize significance of edges but rather only provides an additional metric of stability under resampling. Notably, the majority of edges in the graph existed in a very high percentage of resampled analyses. Somewhat lower stability was found for edges between (as opposed to within) different domains, as expected based on the correlation-based grouping of domains. Interestingly, a small number of edges that were extremely stable in existence had relatively lower directional stability; this suggests (but does not demonstrate) a potential reciprocal relationship between variables that could potentially be elucidated in longitudinal data. For example, the edge between social support and negative affect existed in 100% of resampling analyses, but in many resamples this edge instead went from negative affect to social support, indicating a potential bidirectional relationship.

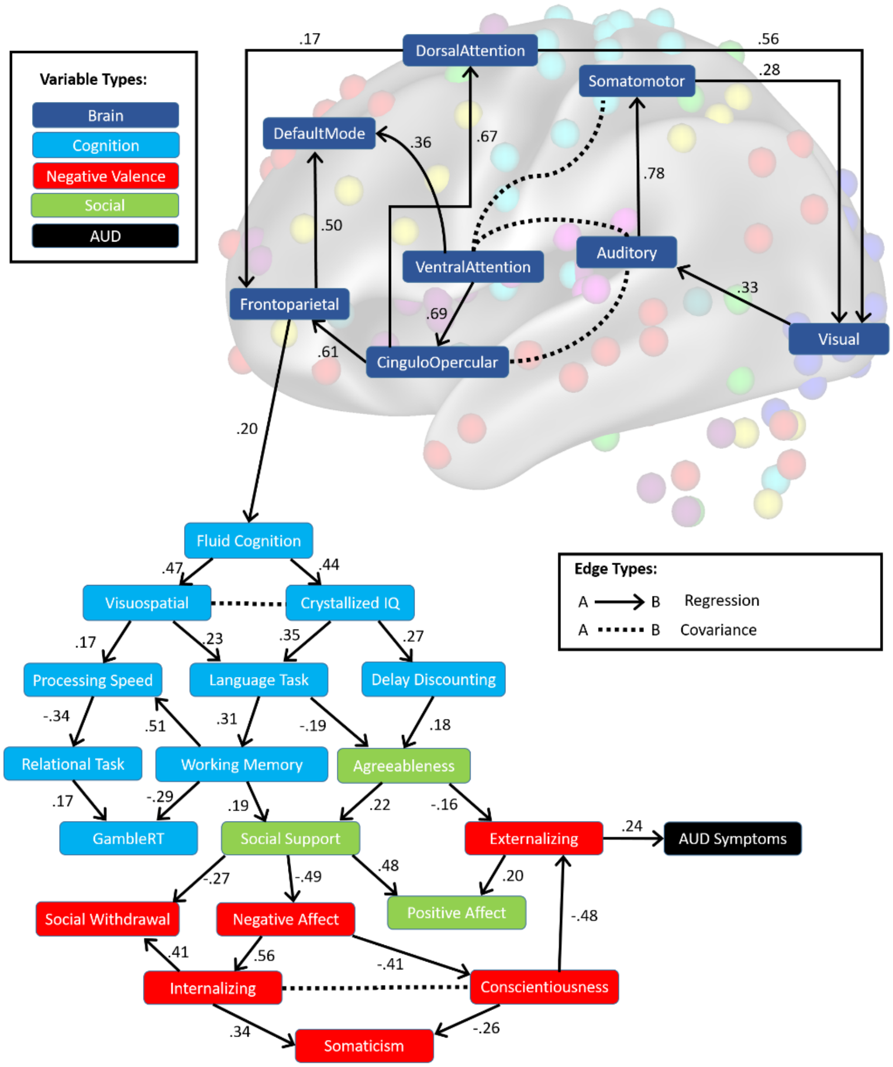

Supplementary Figure S3. We replicated our discovered causal model using a subset of the networks defined in (1). *We fit an a priori structural equation model (SEM) to these alternatively defined networks using the edges discovered in our analysis of the Cole-Anticevic networks. Since the Greene-300 parcellation included only one visual network, while the Cole-Anticevic contained two, we merged the discovered visual1 and visual2 edges for the SEM. Specifically, in the discovered graph [Figure 3], visual2 connectivity was caused by somatomotor and dorsal attention connectivity, and visual1 connectivity caused auditory connectivity; in the replication SEM, we included incoming visual edges from somatomotor and dorsal attention, and an outgoing edge to auditory. Standardized edge weights recovered via SEM are displayed in text next to each edge in the graph. The overall SEM fit was almost as good as in the initial parcellation, RMSEA = .06, TLI = .87.*

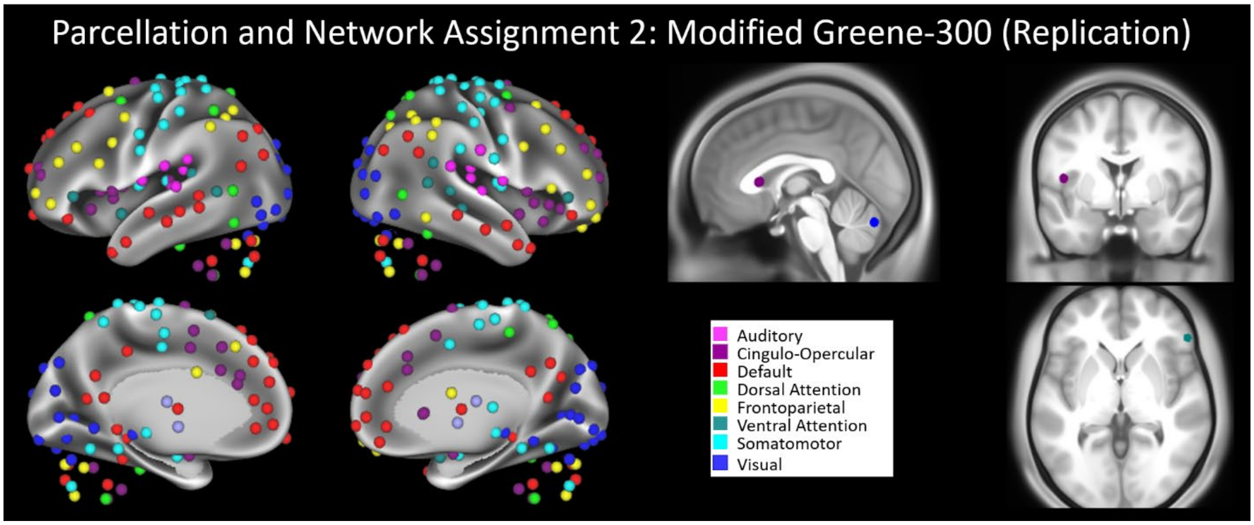

Supplementary Figure S4. To determine whether our main results would replicate in an independently-derived brain network parcellation, we used a slightly modified version of the Greene-300 ROI-based parcellation described in (1)*. As this was intended as a replication analysis, we brought these networks into agreement with the networks from our primary analysis by merging the cingulo-opercular and salience networks into one network, and merging the lateral and dorsal somatomotor network into a single somatomotor network.*

Supplementary Table S2. Correspondence between primary analysis brain networks (2) *and replication networks* (1)*. Correlations represent the correlation of average within-network connectivity values, across subjects.*

Cole-Anticevic	Greene-300	Correlation
Frontoparietal	Frontoparietal	r = .84, p = 3.6e-289
Default Mode	Default Mode	r = .91, p ≈ 0
Dorsal Attention	Dorsal Attention	r = .80, p = 4.5e-237
CinguloOpercular	CinguloOpercular & Salience	r = .86, p ≈ 0
Auditory	Auditory	r = .87, p ≈ 0
Visual2	Visual	r = .93, p ≈ 0
Somatomotor	Somatomotor (Lateral & Dorsal)	r = .95, p ≈ 0
Language	Ventral Attention	r = .86, p ≈ 0
Orbitoaffective	*	*
Ventral Multimodal	*	*
Posterior Multimodal	*	*
*	SOFA	*
*	Medial Temporal	*
*	Parietomedial	*

SUPPLEMENTARY REFERENCES

1. Seitzman BA, Gratton C, Marek S, Raut RV, Dosenbach NUF, Schlaggar BL, et al. A set of functionally-defined brain regions with improved representation of the subcortex and cerebellum. NeuroImage. 2020 Feb 1;206:116290.

2. Ji JL, Spronk M, Kulkarni K, Repovš G, Anticevic A, Cole MW. Mapping the human brain's cortical-subcortical functional network organization. NeuroImage. 2019 Jan;185:35–57.
