# Supplementary material for: An integrated multimodal model of alcohol use disorder generated by data-driven causal discovery analysis
Source: Commun Biol. 2021 Mar 31;4:435. doi: 10.1038/s42003-021-01955-z (PMC8012376; doi:10.1038/s42003-021-01955-z)
Supplement: Supplementary file 3 — Description of Additional Supplementary Files [file 42003_2021_1955_MOESM3_ESM.pdf]

Description of Additional Supplementary Files

File name: Supplementary Data 1
Description: Parallel Analysis to determine number of factors for EFA.

File name: Supplementary Data 2
Description: Factor loadings from EFA of HCP data. Factor loadings >.3 are highlighted for visibility.

File name: Supplementary Data 3
Description: Factor loadings from EFA of HCP data following standardization (z-scoring). Factor loadings >.3 are highlighted for visibility.

File name: Supplementary Data 4
Description: Correlation structure of factors.

File name: Supplementary Data 5
Description: Resampling stability of graph edges.

File name: Supplementary Data 6
Description: List of included and excluded variables.

File name: Supplementary Data 7
Description: Descriptive statistics of the included variables.

File name: Supplementary Data 8
Description: Data used for GFCI Model Fitting and SEM Fitting.
