# Supplementary material for: An integrated multimodal model of alcohol use disorder generated by data-driven causal discovery analysis
Source: Commun Biol. 2021 Mar 31;4:435. doi: 10.1038/s42003-021-01955-z (PMC8012376; doi:10.1038/s42003-021-01955-z)
Supplement: Supplementary file 12 — Reporting Summary [file 42003_2021_1955_MOESM12_ESM.pdf]

nature research

Corresponding author(s): Eric Rawls

Last updated by author(s): Feb 10, 2021

Reporting Summary

Nature Research wishes to improve the reproducibility of the work that we publish. This form provides structure for consistency and transparency in reporting. For further information on Nature Research policies, see our Editorial Policies and the Editorial Policy Checklist.

Statistics

For all statistical analyses, confirm that the following items are present in the figure legend, table legend, main text, or Methods section.

n/a	Confirmed	
☐	☒	The exact sample size (*n*) for each experimental group/condition, given as a discrete number and unit of measurement
☐	☒	A statement on whether measurements were taken from distinct samples or whether the same sample was measured repeatedly
☐	☒	The statistical test(s) used AND whether they are one- or two-sided<br>*Only common tests should be described solely by name; describe more complex techniques in the Methods section.*
☐	☒	A description of all covariates tested
☐	☒	A description of any assumptions or corrections, such as tests of normality and adjustment for multiple comparisons
☐	☒	A full description of the statistical parameters including central tendency (e.g. means) or other basic estimates (e.g. regression coefficient) AND variation (e.g. standard deviation) or associated estimates of uncertainty (e.g. confidence intervals)
☐	☒	For null hypothesis testing, the test statistic (e.g. *F*, *t*, *r*) with confidence intervals, effect sizes, degrees of freedom and *P* value noted<br>*Give P values as exact values whenever suitable.*
☒	☐	For Bayesian analysis, information on the choice of priors and Markov chain Monte Carlo settings
☐	☒	For hierarchical and complex designs, identification of the appropriate level for tests and full reporting of outcomes
☐	☒	Estimates of effect sizes (e.g. Cohen's *d*, Pearson's *r*), indicating how they were calculated

Our web collection on statistics for biologists contains articles on many of the points above.

Software and code

Policy information about availability of computer code

Data collection	Data was provided by the Human Connectome Project S1200 release (2017).
Data analysis	Factor analysis (parallel analysis and factor extraction) was done using the 'psych' package (version 2.0.12) (26), and factor rotation used the 'GPArotation' package (version 2014.11-1) for R (version 3.6.3). FMRI network connectivity analysis was done using Connectome Workbench (version 1.4.2) and MATLAB (version R2018b). Causal discovery analysis (GFCI) was done using Tetrad (version 6.7). Stability analysis (jackknifing) was done in Tetrad (version 6.7) using 90% subsampling and 1000 replications. Effect size calculation was done using the 'lavaan package (version 0.6-6) running in R (version 3.6.3).

For manuscripts utilizing custom algorithms or software that are central to the research but not yet described in published literature, software must be made available to editors and reviewers. We strongly encourage code deposition in a community repository (e.g. GitHub). See the Nature Research guidelines for submitting code & software for further information.

Data

Policy information about availability of data

All manuscripts must include a data availability statement. This statement should provide the following information, where applicable:

- Accession codes, unique identifiers, or web links for publicly available datasets
- A list of figures that have associated raw data
- A description of any restrictions on data availability

The data analyzed for this study are available from the WU-Minn Human Connectome Project Consortium (https://www.humanconnectome.org/study/hcp-young-adult/document/1200-subjects-data-release). This data is available for public use with a data use agreement.

Field-specific reporting

Please select the one below that is the best fit for your research. If you are not sure, read the appropriate sections before making your selection.

☐ Life sciences ☒ Behavioural & social sciences ☐ Ecological, evolutionary & environmental sciences

For a reference copy of the document with all sections, see nature.com/documents/nr-reporting-summary-flat.pdf

Behavioural & social sciences study design

All studies must disclose on these points even when the disclosure is negative.

Study description	Data were collected by the Human Connectome Project over a period of five years (2010-2015). The study used a broad phenotyping approach, to characterize individual differences in a community sample of young adults on as many common self-report and behavioral variables as possible. Subjects also underwent structural and functional MRI scanning, to characterize intrinsic brain connectivity profiles contributing to phenotypic individual differences.
Research sample	Subjects were young adults age 22-35, 54% female. Subjects provided written informed consent at Washington University.
Sampling strategy	Data for the HCP relied on a community sampling strategy, as the goal of the study was to characterize normal brain function, and the relation of brain function to normative individual differences. Subjects with a pre-existing psychiatric diagnosis were excluded, although notable numbers of subjects indicated that they fulfilled clinical criteria for alcohol use disorder.
Data collection	Broad phenotypic data were collected in-person using computerized questionnaire batteries and behavioral tasks, and (f)MRI data were collected on a customized Siemens scanner at Washington University. Alcohol use and symptoms of alcohol use disorder were assessed using a structured interview. All data collection protocols are reported by the HCP, therefore, the data collection for this analysis is extremely well documented.
Timing	HCP data was collected over 5 years (2010-2015), and the final data release occurred in 2017.
Data exclusions	The only exclusion strategies applied were to exclude participants with missing data in any of the measures of interest. The decision was made a priori to exclude participants with missing data rather than to impute data, since the HCP dataset is high-quality and very little data is missing.
Non-participation	All data released as part of the HCP reports exactly which participants completed each portion of the study, therefore non-participation is extremely well documented. Out of ~1200 subjects enrolled, we were able to retain 926 subjects with complete data in all modalities, representing a high-quality sample given the high number of measures each subject completed.
Randomization	Participants were not randomized into experimental groups.

Reporting for specific materials, systems and methods

We require information from authors about some types of materials, experimental systems and methods used in many studies. Here, indicate whether each material, system or method listed is relevant to your study. If you are not sure if a list item applies to your research, read the appropriate section before selecting a response.

Materials & experimental systems

n/a	Involved in the study	
☒	☐	Antibodies
☒	☐	Eukaryotic cell lines
☒	☐	Palaeontology and archaeology
☒	☐	Animals and other organisms
☐	☒	Human research participants
☒	☐	Clinical data
☒	☐	Dual use research of concern

Methods

n/a	Involved in the study	
☒	☐	ChIP-seq
☒	☐	Flow cytometry
☐	☒	MRI-based neuroimaging

Human research participants

Policy information about studies involving human research participants

Population characteristics	See above
Recruitment	Subjects were recruited from Missouri families, based on state records from the Missouri Department of Health and Senior Services Bureau of Vital Records. Recruiting was specifically focused on retaining a sample that reflected the overall demographic makeup of the U.S.
Ethics oversight	All protocols were approved by the Washington University IRB.

Note that full information on the approval of the study protocol must also be provided in the manuscript.

Magnetic resonance imaging

Experimental design

Design type	Resting-state
Design specifications	1200 frames collected in four sessions of 14 minutes:33 seconds for each participant; we included all subjects who had at least one full day of data (two sessions).
Behavioral performance measures	None (resting-state)

Acquisition

Imaging type(s)	functional, structural
Field strength	3T
Sequence & imaging parameters	Functional: gradient-echo EPI, 720 ms TR, 33.1 ms TE, 52 degree flip angle, 208x180 mm FOV, 104x90 matrix, 2mm slice thickness, 72 slices, 2 mm isotropic voxels, multiband factor = 8, echo spacing = .58 ms, BW = 2290 Hz/Px<br>T1 Structural: TR=2400 ms, TE = 2.14 ms, TI = 1000 ms, flip angle = 8 degrees, 224x224 FOV, 0.7 mm isotropic voxels, BW = 210 Hz/Px
Area of acquisition	whole-brain

Diffusion MRI ☐ Used ☒ Not used

Preprocessing

Preprocessing software	FSL, Connectome Workbench, and Freesurfer.
Normalization	Nonlinear (volume) using FSL, surface coregistration using Freesurfer.
Normalization template	2mm MNI template
Noise and artifact removal	Data were cleaned of artifacts using regression of 24 physiological measures and further cleaned using a machine learning approach (ICA-FIX).
Volume censoring	Previous analyses by the HCP team found that ICA-FIX resulted in higher quality data than volume censoring, so no volume censoring was used.

Statistical modeling & inference

Model type and settings	fMRI network connectivity was calculated by averaging z-transformed Pearson correlation values (ROI-to-ROI) within each of 12 predefined, data-derived networks.
Effect(s) tested	No effects tested - 12 network connectivity values (average) were used as individual difference factors.

Specify type of analysis: ☒ Whole brain ☐ ROI-based ☐ Both

Statistic type for inference (See Eklund et al. 2016)	N/A
Correction	N/A for resting-state fMRI analysis

Models & analysis

n/a	Involved in the study
☐	☒ Functional and/or effective connectivity
☒	☐ Graph analysis
☐	☒ Multivariate modeling or predictive analysis

Functional and/or effective connectivity	Pearson correlation, z-transformed to approximate normal distribution
Multivariate modeling and predictive analysis	Dimension reduction on phenotypic variables used maximum likelihood factor analysis, and whole-brain fMRI measures used average within-network connectivity within each of 12 a priori, data-derived networks of interest. Machine learning analysis used Tetrad v6.7.0 with the GFCI algorithm. The best causal model was defined using Bayesian Information Criteria, and model fit was assessed using structural equation modeling (RMSEA, TLI) indices.
